# Robust host source tracking building on the divergent and non-stochastic assembly of gut microbiomes in wild and farmed large yellow croaker

**DOI:** 10.1186/s40168-021-01214-7

**Published:** 2022-01-26

**Authors:** Jun Zhu, Hao Li, Ze Zhou Jing, Wei Zheng, Yuan Rong Luo, Shi Xi Chen, Feng Guo

**Affiliations:** 1 State Key Laboratory of Cellular Stress Biology, School of Life Sciences, Xiamen University, Xiamen, China; 2 Southern Marine Science and Engineering Guangdong Laboratory (Zhuhai), Zhuhai, China; 3 State-Province Joint Engineering Laboratory of Marine Bioproducts and Technology, College of Ocean and Earth Sciences, Xiamen University, Xiamen, China; 4 Key Laboratory of the Ministry of Education for Coastal and Wetland Ecosystems, Xiamen University, Xiamen, China

## Abstract

**Background:**

Given the lack of genetic background, the source tracking unknown individuals of fish species with both farmed and wild populations often cannot be robustly achieved. The gut microbiome, which is shaped by both deterministic and stochastic processes, can serve as a molecular marker of fish host source tracking, particularly as an alternative to the yet-to-be-established host genetic marker. A candidate for testing the feasibility is the large yellow croaker, *Larimichthys crocea*, which is carnivorous and ranks the top mariculture fish in China. Wild resource of this fish was depleted decades ago and might have potential problematic estimation because of escaping of farmed individuals.

**Results:**

The rectums of wild (*n* = 212) and farmed (*n* = 79) croakers from multiple batches were collected for the profiling of their gut bacterial communities. The farmed individuals had a higher alpha diversity and lower bacterial load than the wild individuals. The gut microbiota of the two sources exhibited divergence and high inter-batch variation, as featured by the dominance of *Psychrobacter* spp. in the wild group. Predicted functional capacity of the gut microbiome and representative isolates showed differences in terms of host source. This difference can be linked to the potential diet divergence between farmed and wild fishes. The non-stochastic distribution pattern of the core gut microbiota of the wild and farmed individuals supports the feasibility of microbiota-based host source tracking via the machine learning algorithm. A random forest classifier based on the divergence and non-stochastic assembly of the gut microbiome was robust in terms of host source tracking the individuals from all batches of croaker, including a newly introduced batch.

**Conclusions:**

Our study revealed the divergence of gut microbiota and related functional profiles between wild and farmed croakers. For the first time, with representative datasets and non-stochastic patterns, we have verified that gut microbiota can be robustly applied to the tracking of host source even in carnivorous fish.

Video abstract

**Supplementary Information:**

The online version contains supplementary material available at 10.1186/s40168-021-01214-7.

## Introduction

Microbes living in animal gastrointestinal tracts play important roles in the nutrition and health of their hosts through extensive metabolic and immune interactions [1–4]. With the development of next-generation sequencing techniques, the diversity and function of the gastrointestinal microbiomes in many fish species have been unprecedentedly explored in the last decade [1, 5, 6]. Fish usually have much more dynamic and less diverse gut microbiomes than terrestrial vertebrates [7]. A variety of factors, such as source (i.e., domesticated or wild), habitat, diet, size, life-stage or age, and geographic origin, have a strong impact on the microbiomes in fish gut [5, 8–10] Moreover, herbivorous and omnivorous fishes usually have higher selectivity in their intestine compared with carnivorous fish [11, 12], while host selectivity in certain microbial taxa has also been reported for carnivorous Atlantic salmon [13].

Among the gut microbiota, core microbial taxa, which can be detected in most or all host individuals, can help to elucidate the patterns of physiological interactions and evolutionary relationships between microbes and hosts [6, 12, 14, 15]. In most of those studies, the core taxa were proposed on the basis of limited sampling batches despite the high dynamics of fish gut microbiota. From an ecological point of view, the assembly of microbiomes is underpinned by two factors: deterministic and neutral (or stochastic) processes [16–18]. Reasonably, core taxa are more likely to be selected by deterministic factors (e.g., certain host-specific factors, [19]) than by stochastic processes. Deciphering the dominant factor is a basis for the further examination of specific host–microbe relationships or potential applications.

Understanding the divergence of gut microbiota between conspecific wild and farmed fishes can contribute to the improvement of diet efficiency, farming mode, and probiotics development [20–24]. Moreover, the divergence of gut microbiota between wild and farmed fishes may be informative for host source tracking. The continuously increasing production of mariculture fish species and the interaction between farmed and wild fishes [25, 26], coupled with divergent selling price, risk of food safety [27–29], have accentuated the need of host source tracking. Another possible application is to discriminate fishes escaping from farmed cages and seedling release individuals from true wild individuals. For fish species with sufficient historical specimens and genetic background, such as salmon, the genetic marker from the host showed good performance in terms of source tracking [30]. However, for most mariculture fish species with poor genetic markers requiring an identification of wild and farmed populations, the gut microbiome, as the secondary genome of corresponding host, can serve as an alternative biomarker of host source.

The large yellow croaker *Larimichthys crocea* (hereafter called “croaker”) is an economically important marine carnivorous fish species in China [31]. Long-term overfishing since the 1950s has resulted in the severe depletion (> 95%) of wild stocks. Currently, the majority of sales is from mariculture, exceeding 220,000 tons in 2019 (the top mariculture fish in China, [32]). Wild stock enhancement through the release of tens of millions of fry has been performed annually for over a decade. However, the yield from the wild stock continued to show minimal increase, possibly because of fishing pressure, human interference on habitats, niche occupation by mariculture, loss of genetic diversity, and poor adaptation of released juveniles [33]. Moreover, wild resources are likely overestimated. Croakers captured in coastal regions may overlap with or are adjacent to the mariculture region of the species; in other words, the wild resources may be directly derived from escaped farmed individuals. Given the insufficient accumulation of “true” wild individuals, to our knowledge, the host genetic biomarkers that can be used to distinguish wild croakers from domesticated ones have not been well established [34].

The divergence of gut microbiomes between wild and farmed croakers has not been characterized. For carnivorous species with multiple geographic populations [35], the inter-batch variations may be high in their gut microbiota. Therefore, this species seems to be a suitable candidate for testing the feasibility of microbiota-based host source tracking. In this study, we profiled the bacterial community and core taxa obtained from the rectums of farmed and wild croakers. The croaker specimens were sampled from various geographical populations and batches. First, we confirmed the divergence between the gut microbiome of wild and farmed fishes in terms of the alpha- and beta-diversity. Then, the fitness of the neutral model was evaluated, and a classifier was established using the random forest model. Finally, a machine learning approach that is suitable for unbalanced distribution data with noise features and less prone to overfitting [36, 37] was implemented, and the feasibility of using the divergence of the microbiota between captive and wild individuals in source tracking was verified.

## Materials and methods

### Sample collection and preparation

Ten batches (designated as A, B, C, F, H, N, S, T, W, and X; *n* = 291) of croakers from diverse locations and of different sizes were collected from wild catching (C, F, S, and W; *n* = 212) and raft farming sites (A, B, H, N, T, and X; *n* = 79). The details of the sampling sites and the batches are shown in Fig. S1. Batch S, a unique wild batch collected from a bay with a high-density mariculture of croakers, was only used to test the host source tracking classifier. Notably, large wild individuals (> 300 g) are difficult to obtain because their natural stocks have depleted since the 1980s. Farmed fishes were mainly fed with artificial formulated feed and occasionally with fresh fish meals during the sampling period. All individuals were frozen at − 20 °C immediately after they were removed from seawater. In this study, we sampled the gut microbiome of the rectum section (also called hindgut) for the future application of the non-invasive sampling strategy (e.g., by inserting a swab through the anus). Approximately 1–2 cm of the rectum was aseptically removed from the abdominal cavity by using sterile scissors and tweezers (see Fig. S2 for the details of the digestive organs and the typical sampled section). Then, the tissue samples were transferred to centrifuge tubes and stored at − 20 °C prior to the DNA extraction.

### DNA extraction

The DNA extraction was performed using a commercial kit (QIAamp PowerFecal DNA Kit, QIAGEN, Germany). Before extraction, the tissues with rectum contents were aseptically homogenized with a tissue homogenizer after adding 200 μL of Solution CD1 (a buffer of the kit). Both the rectum and content were processed together for DNA extraction. Then, by using MilliQ water as the extraction blank, the DNA extraction was performed according to kit instructions. To minimize DNA contamination from the extraction buffers, we used freshly prepared MilliQ water to elute the DNA in the final step. After extraction, the quantity and quality of the yielded DNA were examined with a micro-spectrophotometer (NanoDrop ND-1000, Thermo Scientific, USA). The OD_260nm_/OD280_nm_ ratios ranged from 1.7 to 2.0 for all samples.

### PCR and high-throughput sequencing

The PCR targeting the V4 region of the bacterial 16S rRNA gene was conducted according to a previously described method [38] except for the addition of adaptor sequences during library construction. To minimize potential cross-talking contamination, as suggested by a previous study [39], we applied unique barcodes to link the forward and reverse primers during multiplexing (i.e., no barcode was shared by any sample in a library). The number of PCR cycles was set to 30, under which the DNA extraction blank and PCR blank (MilliQ water) did not produce visible bands during electrophoresis. The purified PCR products were pooled together at equal mass before sequencing for library construction (TruSeq DNA PCR-Free Library Preparation Kit, Illumina, USA). Then, a high-throughput sequencing was performed in the Illumina Hiseq2500 sequencing platform with the PE250 strategy (commercial service provided by Novogen, China).

### Quantitative PCR for determining bacterial load

To determine the bacterial load in the rectum, randomly picked 20 samples from both wild (batches C and F) and farmed (batches H and T) were analyzed by quantitative PCR (qPCR). Due to the relatively long length of the V4 amplicon, the region of V3 was amplified using the primer sets of 341F and 534R [40]. For each sample, 5 ng of the DNA template was added to 25 μL of PCR solution (final volume, SYBR GreenER™ qPCR SuperMix Universal, ThermoFisher Scientific, USA). A standard curve (*R*^2^ > 0.99) generated by the 10-fold dilutions of a plasmid DNA containing a full-length 16S rRNA gene from *Escherichia coli* was used in absolute quantification. qPCR was performed in triplicate for each sample. To calculate the bacterial load per unit of host tissue, by referring to the standard curve, we quantified 16S rRNA gene copy number per ng of DNA because most of the extracted DNA was derived from the host tissue (indicated by the low copy number of bacterial 16S rRNA gene per ng DNA). The difference in bacterial loads between the farmed and wild samples was determined by the Wilcoxon test.

### Analysis of 16S rRNA gene high-throughput sequencing data

Raw high-throughput sequencing data were cleaned using TRIMMOMATIC [41]. USEARCH v10 was used to remove the suspicious sequences (i.e., chimeras and rare sequences with frequency of less than 8 across all samples) and determine the 0.97-level operational taxonomic units (OTUs) by means of the UNOISE algorithm and UPARSE, respectively [42, 43]. Then, the table of OTU abundance generated in the USEARCH platform was introduced into Mothur v1.39.5 for alpha-diversity and beta-diversity analyses and taxonomic classification [38]. Data normalization was performed by subsampling 10,000 valid readings for each sample [38]. For the beta-diversity analysis, the weighted Unifrac distance was calculated following the Mothur Miseq SOP [44]. A Wilcoxon test was conducted to compare the alpha- and beta-diversity analyses. We used the EzBioCloud 16S database as the taxonomic reference [45]. The effect of four factors (body weight, season, source, and batch) on the bacterial community was estimated using analysis of variance [46]. We conducted an analysis of molecular variance (AMOVA) in Mothur to determine the significance of inter-group differences among community structures. The heatmap with the sample and OTU-level clustering was realized in R code by using the *pheatmap* v1.0.10 and *vegan* v2.5-3 packages [47, 48]. Given the high inter-batch variation, the core microbiota at the OTU level was defined as the taxa detected in > 70% individuals for wild or farmed samples.

To determine the importance of the stochastic process in the assembly of gut microbiomes, the Sloan neutral model was tested using the R v3.5.1 code [16, 49]. Additionally, the relative importance of stochastic and deterministic processes in the community assembly, nearest taxon index (NTI), and beta nearest-taxon index (βNTI) were calculated using the *Picante* v1.8.2 and *MicEco* v0.9.15 R package (OTUs: abundance > 0.01%, abundance.weighted = True) [50, 51]. The 16S rRNA gene-based MetaCyc pathway profiling were inferred using PICRUST2, and the differently abundant MetaCyc pathways of the farmed and wild croakers were identified using the *ALDEx2* v1.14.1 R package [52, 53].

### Isolation of typical gut bacteria and genome analysis

Three farmed (batch T) and three wild (batch C) individuals were used for gut bacterial isolation. The microorganisms in freshly prepared rectums (~ 0.5 g) were rigorously washed off prior to serial 10-fold dilution in sterile 0.9% NaCl. Then, the dilutions were spread on 2216E agar plates (Hope Bio-Technology Co., Ltd., Qingdao, China) and cultivated for 48 h at 20 °C. The colonies with different morphologies and colors were selected, and the respective taxonomy was determined via full-length 16S rRNA gene sequencing. Only the isolates affiliated with *Photobacterium* (*n* = 7) and *Psychrobacter* (*n* = 7), which form the representative taxa of farmed and wild croakers, respectively, were kept for downstream analysis.

The genomic DNA of the isolates was extracted and sequenced using the Illumina HiSeq X Ten platform. An assembly was performed with SPAdes v3.9.0 (parameters: -t 50, -k 55, 77, 99 -careful) [54]. Only the scaffolds of > 1000 bp were used to predict the open reading frames by using Prodigal v2.6.3 [55]. The carbohydrate-active enzyme (CAZyme) families were annotated using dbCAN2 v2.0.11 under default parameters, and the signal peptides were predicted using SignalP v4.0 [56, 57]. The optimal pH of the CAZymes was predicted using the AcalPred online server [58]. The capability of organic acid production of the *Photobacterium* and *Psychobacter* isolates was annotated using DRAM [59].

### Assays of pH measurement for rectum content and bacterial biofilm formation

The pH of the rectums of croakers was measured by transferring each freshly prepared rectum (~ 0.5 g; containing content) from the wild (*n* = 27) and farmed (*n* = 15) individuals into a 15-mL centrifuge tube and gently and thoroughly washing the specimens in 5 mL ddH_2_O. Then, the pH values of the suspensions were measured using a pH measurer. To test the capability of biofilm formation of the isolates, we inoculated each of the isolates into replicate wells (*n* = 6) of a 96-well plate containing 200 μL of 2216E broth (approximately 10^6^ cells per well). After growth for 48 h, the OD_600_ of the cell suspension was measured with a microplate reader (CLARIO Star® Plus, BMG LABTECH Inc., USA). Then, the biofilm was stained with 0.1% crystal violet, and the OD_550_ of the ethanol elution was measured [60]. The OD_550_/OD_600_ ratio was used to determine the capability of biofilm formation. The images of the stained biofilms were recorded under an inverted light microscope.

### Random forest classification for wild and captive individuals

To distinguish the wild and farmed individuals via the machine learning approach, we used the random forest algorithm to construct a classifier. The dataset (nine batches except for batch S, *n* = 276) was pre-processed by removing the rare OTUs (< 20% frequency). The samples were split into two partitions, namely the training and testing datasets, under different proportions with 10 iterations. We built the classifier with the *random forest* v4.6.14 R package with 5001 trees and default *mtry* number on the training samples and then validated it on the test samples [61]. The receiver operating characteristic curve was obtained via the *pROC* v1.16.2 R package [62]. The top 15 OTUs in terms of mean decrease in accuracy were used to rebuild an optimized classifier, and the accuracy of the model was accessed via leave-one-out cross-validation by using the *caret* v6.0.86 R package [63]. We also tested the reliability of the classifier by using new wild samples (batch S, *n* = 15) that were caught in a bay where the farmed individuals of A, B, H, T, and X were collected (Fig. S1).

## Results

### Divergence of bacterial alpha diversity, abundance, and high-rank taxa in the rectums of wild and farmed croakers

**Fig. 1:**
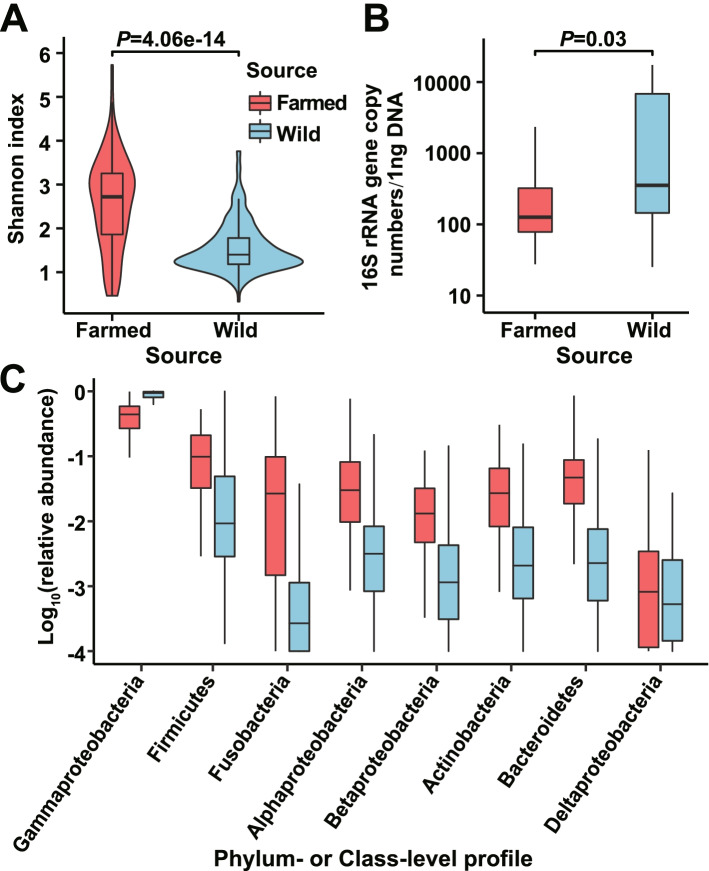
Bacterial alpha diversity (**A**), load (**B**), and high-rank taxa (**C**) in the rectum of the wild and farmed croakers. Wilcoxon test was applied to the comparisons

Given a sampling depth of 10,000 sequences, the Shannon index of the bacterial community in the rectums of the croakers ranged from 1.17 to 4.96, with a median value of 3.80. Interestingly, the wild individuals presented a lower diversity than the farmed ones (Fig. 1A, *P* = 4.06e^−14^, Wilcoxon test). As the copy number of 16S rRNA gene per ng DNA for a putative bacterium with 4M genome and four copies of 16S rRNA gene was 9.2×10^5^, the determined 16S rRNA gene copy number (only 9–17,409 copies per ng DNA, Fig. 1B) indicates a low bacterial load in the rectums of the wild and farmed individuals. However, the wild individuals contained more gut bacterial inhabitants than the farmed ones (Fig. 1B, *P* = 0.03, Wilcoxon test). The major detected bacterial phyla (or classes of *Proteobacteria*) were *Gammaproteobacteria*, *Firmicutes*, *Fusobacteria*, *Alphaproteobacteria*, *Betaproteobacteria*, *Actinobacteria*, *Bacteroidetes,* and *Deltaproteobacteria*. A significant difference (*P* < 0.05, Wilcoxon test, false discovery rate (FDR)-corrected *P* value) between the wild and captive individuals was observed for the relative abundance of nearly all of the abovementioned high-rank taxa (Fig. 1C). Dominance of *Gammaproteobacteria* was detected in both wild and farmed samples (median percentage: 93.9% and 44.0%).

### Beta-diversity indicated batch- and source-associated variation of gut microbiota

**Fig. 2:**
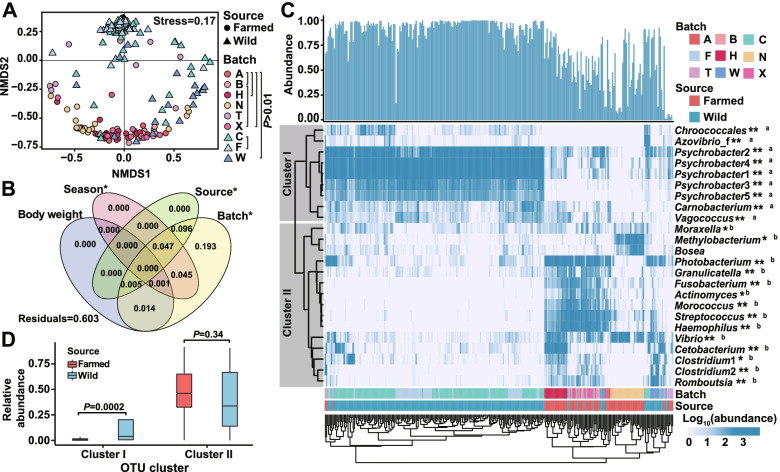
Beta-diversity of the gut microbiota of croakers. **A** NMDS carried out on the weighted Unifrac distance between the farmed and wild groups. Only inter-batch pairs without significant differences are marked (AMOVA test, *P* > 0.01). **B** The explanation of variance in gut microbiomes by batch, source, sampling season, and body weight (*P < 0.05). **C** Heatmap shows the main OTUs relative abundance in the farmed and wild individuals. Only 24 OTUs with the mean abundance of > 0.2% and occurrence of > 20% are shown. Rows are clustered according to Pearson correlation, and OTUs are stratified into two clusters. The columns (samples) are clustered according to Euclidean distance. The total relative abundance of individuals is shown in the bar plot. Difference of the relative abundance for each taxon between farmed and wild samples was marked aside the taxon name (a and b for statistically higher in the wide and farmed samples, respectively; *P < 0.05, **P < 0.001, FDR-corrected Wilcoxon test) (D) Relative abundance of OTUs belonging to Clusters I and II in the farmed (*n* = 76) individuals and wild (*n* = 26) ones that are clustered with the majority of the farmed group

The findings from the nonmetric multidimensional scaling (NMDS) showed that the wild and farmed groups could be distinguished to a great extent with a few exceptions (Fig. 2A). The AMOVA results showed a significant divergence between the two sources and among most batches (Fig. 2A). The beta-diversity divergence followed the order: between sources > between batches > within batches (Fig. S3). The divergences among farmed batches were higher than those among wild batches (Fig. S3). Batch, source, and sampling season can explain 0.397, 0.148, and 0.093 of the microbiota variation (all *P* < 0.01), whereas body weight can barely explain the variation (0.020, *P* > 0.05). In fact, all of the abovementioned explainable variations can be explained by batch since source and season have no independent contribution (Fig. 2B, individuals in one batch had consistent source and season property). The high inter-batch divergence suggests the unpredictable overall microbiota variation of the newly introduced batch, which may have a negative impact on reliable host source tracking practice.

The heatmap is shown in Fig. 2C. Twenty-four major gut bacterial OTUs that were present in more than 20% of the samples with over 0.2% mean relative abundance were used in the analysis of the divergence of microbiota. These OTUs accounted for 1.4–99.9% (83.4% in median value) of the total bacterial community in all of the samples. Similar to the NMDS results, although most individuals from each source tended to cluster together, some samples in the wild batch C (*n* = 7), F (*n* = 1), and all individuals of batch W (*n* = 18) were clustered with most (> 95%) farmed individuals. After clustering based on Pearson correlation, the 24 OTUs fell into two clusters, namely Cluster I (9 OTUs, which were enriched in the wild individuals) and Cluster II (15 OTUs, which were generally enriched in the farmed ones). In Cluster I, five *Psychrobacter* OTUs constituted 93.0% (median value) of the total bacteria in the wild samples, whereas OTUs affiliated with *Photobacterium*, *Vibrio*, *Streptococcus*, *Fusobacterium*, and *Clostridium* were the representative taxa of the farmed individuals.

The NMDS and clustering results indicate that the overall profile of the bacterial community of some wild samples was close to those of major farmed fishes. Thus, we further examined the relative abundance of the OTUs of Clusters I and II in these samples (*n* = 26, from C, F, and W) and the clustered farmed samples (*n* = 76, excluding the other six that were clustered with wild samples). As shown in Fig. 2D, in the Cluster I OTUs (sum together), the wild samples have a higher abundance than the farmed samples (*P* < 0.001, Wilcoxon test), whereas no difference can be observed for the Cluster II OTUs. This result indicates that these wild samples still enriched the Cluster I taxa despite their similar microbiota with the farmed ones. These taxa may serve as indicators for host source tracking.

### Distinct functional capability of gut microbiomes in farmed and wild fishes and its potential linkage with diet

**Fig. 3:**
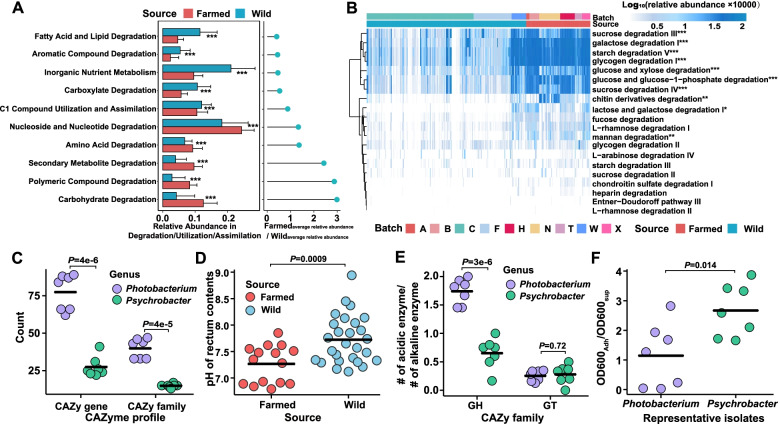
The functional prediction of the gut microbiome and representative isolates belonging to *Photobacterium* and *Psycrobacter*. **A** Significantly differentiated MetaCyc pathways in farmed or wild individuals and only the top 10 pathways with the largest differences are shown. **B** Heatmap shows the relative abundance of MetaCyc pathways (top 20 pathways based on average relative abundance) in farmed and wild samples. **C** The pH of rectum contents in the farmed (*n* = 15) and wild (*n* = 27) individuals. **D** The distribution of CAZyme families and genes in the representative isolates. **E** The ratio of predicted acidic and alkaline glycoside hydrolases and glycosyltransferases in the genome. **F** Biofilm formation capability of *Photobacterium* and *Psycrobacter* isolates. The Welch’s *t*-test (Benjamini–Hochberg-corrected *P* values, *P < 0.05, **P < 0.01, ***P < 0.001) was used in (**A**) and (**B**). Wilcoxon test was used in (**C**), (**D**), (**E**), and (**F**)

The underlying mechanism responsible for the divergence of gut microbiota between wild and farmed croakers was determined by initially predicting the function of the microbiomes by using PICRUSt2. As expected, the functional microbiomes of the wild samples were significantly different from the farmed ones (*P* < 0.001, AMOVA, Fig. S4). The dissimilarity between the pathway and microbiota was highly correlated (Fig. S4). In the top-level functional catalogue, the relative abundance of the degradation/utilization/assimilation-related pathways corresponds to a significant difference between the wild and captive groups (Fig. S5). The relative abundance of the second-level catalogues in this category also exhibited a high divergence between the two groups (Fig. 3A). Remarkably, the wild samples were enriched by fatty acid and lipid degradation pathways, whereas the farmed samples contained higher proportions of carbohydrate and polymeric compound degradation pathways. The pathway-level profiling indicates that the functions enriched in the farmed samples were related to the degradation of starch, glycogen, chitin, mannan, glucose, galactose, etc. (Fig. 3B).

The functional prediction of the microbiomes suggests that diet is a potential causation of the divergence of gut microbiota between the farmed and wild croakers. The farmed croakers are typically fed with formulated food containing high proportions and diverse sources of carbohydrates (~ 30% in dry weight from starch, soybean meal, shrimp meal, and yeast; see Fig. S6 for the typical diet content). However, in natural habitats, croakers usually prey on zooplankton (mostly crustaceans) and small fishes [64] that contain few carbohydrates, although they may obtain high-level chitin-like materials from crustaceans.

To further examine the hypothesis in which diet is a deterministic factor of the microbiota divergence of the wild and farmed croakers, we analyzed the genomes of 14 representative isolates affiliated with *Photobacterium* (Ph1 to Ph7) and *Psychrobacter* (Ps1 to Ps7) that were obtained from the farmed and wild samples, respectively. The quantification of linking 16S rRNA genes from the isolates and the V4 OTUs (requiring > 97% similarity) showed that *Photobacterium* isolates account for 7.7% and 0.3% (median values) of the total bacteria in the farmed and wild samples, respectively, whereas *Psychrobacter* isolates represent 93.3% and < 0.1% (median value) of the total bacteria in the wild and farmed samples, respectively. The corresponding phylogenetic information is shown in Fig. S7. First, we compared the CAZymes in the isolates of *Photobacterium* and *Psychrobacter*. As expected, *Photobacterium* genomes encoded more CAZyme families and genes than each of the *Psychrobacter* genomes (Fig. 3C). However, chitinases are widely detected in the *Photobacterium* strains but are absent in the *Psychrobacter* strains (Fig. S8). Notably, the genome of croaker can encode three chitinases [65], which may minimize the niche selection for chitin-utilizing microorganisms. Second, fatty acid production was also predicted in the genomes of all of the isolates (Fig. S8). A reasonable assumption is that a high-carbohydrate diet may decrease the pH in the rectum by producing short-chain fatty acids [48]. Thus, we tested the pH values of the rectum contents obtained from the farmed and wild individuals and found that the assumption can be positively supported (Fig. 3D). Third, the prediction of optimal pH for the CAZymes indicates higher proportions of acidic glycoside hydrolases (GHs) in *Photobacterium* than in *Psychrobacter* (Fig. 3E). The difference in the proportions is consistent with the dominant distributions in the guts of the farmed and wild croakers, respectively, although glycosyltransferases (GTs), which are usually involved in polysaccharide biosynthesis, have no such signal (Fig. 3E). Meanwhile, signal peptides were predicted in 50 to 64.8% of the acidic CAZymes of *Photobacterium* genomes, suggesting that most of these enzymes are secreted or bound to the cell surface and may partially function in extracellular environments.

In addition, we tested the biofilm formation capability of 14 isolates. The results indicate that *Psychrobacter* isolates usually form denser biofilms than the *Photobacterium* strains (Fig. 3F), as confirmed microscopically (Fig. S9). Although the experiment was performed in vitro, the result could reasonably explain the higher gut bacterial load of wild croakers than that of the farmed ones (Fig. 1B).

### Major and core taxa follow non-stochastic pattern in farmed and wild croakers

**Fig. 4:**
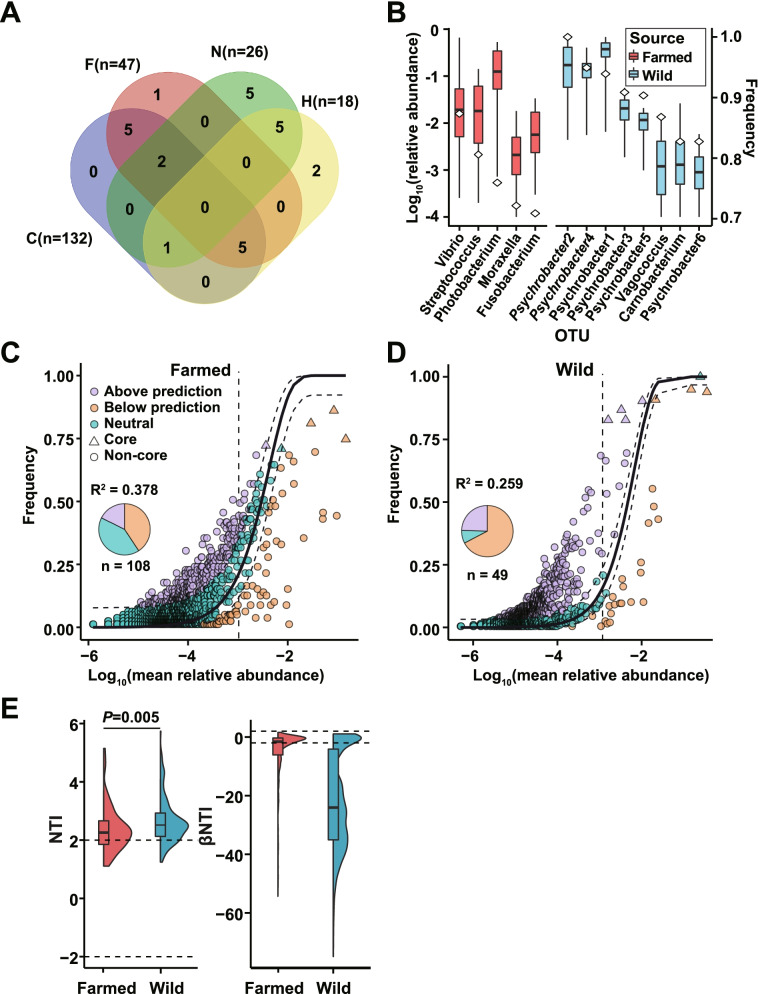
Distribution of the core OTUs and the goodness-of-fit to neutral model for the gut microbiota. The OTUs presented in > 70% samples were defined as core taxa. Core taxa were determined in each of four batches (**A**) and all individuals from each source (**B**). The boxplot shows the relative abundance (left axis) and diamonds are represented detected frequency (right axis) in farmed and wild samples (**B**). The goodness-of-fit to the neutral model for the farmed (**C**) and wild samples (**D**). The dashed curve (in **C** and **D**) represents 99% confidence intervals of the model prediction. *R*^2^ indicates the goodness-of-fit to the neutral model. Pie charts show the proportion of within, above, and below the prediction of high-abundance OTUs (relative abundance > 10^−3^ as separated by the vertical dash line). **E** Violin plot of nearest taxon index (NTI) and beta nearest taxon index (βNTI). Horizontal dashed lines (NTI or βNTI values at − 2 and 2) indicate thresholds for determining the assembly pattern [66].

Although high beta-diversity among batches and sources was detected, this may mainly result from neutral processes other than the deterministic factors. To evaluate the influence of the non-stochastic process on the assembly of gut microbiota in the farmed and wild croakers, we first determined the core taxa that were defined and detected in at least 70% of the samples. Among the C, F, N, and H batches with sufficient individuals, no shared core taxa can be found (Fig. 4A), indicating the high dynamics between sources and among batches as presented previously. After combining the batches, seven and five core OTUs passed the frequency criterion for the wild and farmed sources, respectively (Fig. 4B). *Psychrobacter* OTUs, as the major differential taxa between the farmed and wild samples (Fig. 2C), were the major core taxa for the wild group. By contrast, the core taxa for the farmed fishes were affiliated with *Vibrio*, *Streptococcus*, *Photobacterium*, etc., without any *Psychrobacter* OTU.

Then, all OTUs were examined for their goodness-of-fit to the neutral model for the farmed and wild individuals (Fig. 4C and D, respectively). The values indicate a low goodness-of-fit to the model for both groups (*R*^2^ = 0.378 and 0.259 for the farmed and wild groups, respectively). For the OTUs with a high relative abundance (> 0.1%, mean value), 41.7% for the farmed group fell into the 99% confidence interval, whereas only 8.2% in the wild groups were within this region. Moreover, most core OTUs deviated from the 99% confidence interval except one in the farmed group and one in the wild group (Fig. 4C and D, respectively). The mean NTIs were higher than zero in the farmed and wild individuals (*P* < 0.05), indicating that the phylogenetic relatedness of the microbial taxa in the two communities is more related than expected by chance (Fig. 4E). The βNTI values of 79.8% and 46.1% of the samples were lower than − 2 in the wild and farmed groups, respectively. This finding indicates that deterministic processes (homogeneous selection) are important in the gut microbiome assembly of both wild and farmed croakers [66], although stochastic processes may also play a major role in the community assembly of farmed individuals (Fig. 4E). The results further indicate that non-stochastic processes dominate the assembly of the major and core taxa in the guts of croaker, especially the wild ones. This aspect is fundamental in the application of microbiota-based host source tracking because stochastically assembled communities may introduce more unpredictable noise for newly introduced samples.

### Robust microbiota-based host source tracking based on random forest classification

**Fig. 5:**
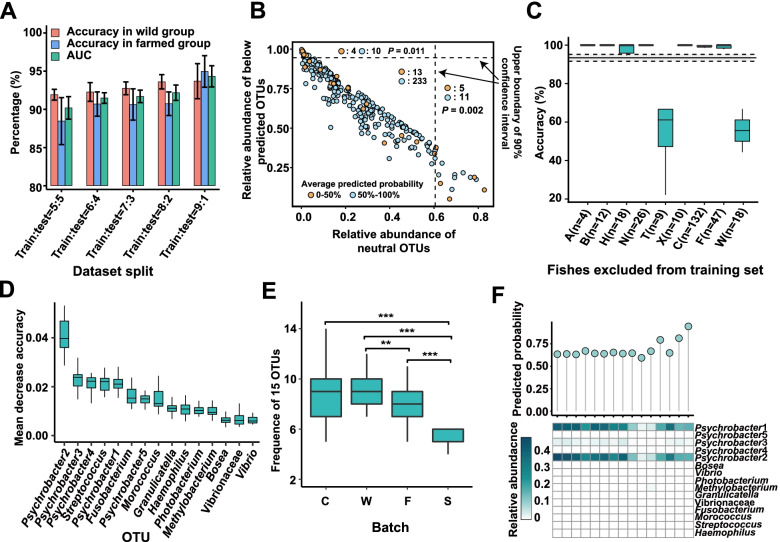
Performance of the random forest classifier. **A** The AUC and predicted accuracy of the farmed and wild groups under different dataset stratifications with 10 replications. **B** Effect of the abundances of OTUs within and below prediction (see Fig. 4**C** and **D** for the definition of the OTUs) on host source tracking. The training set: test set is 5:5 and the bootstrapping number is 100. The dashed line indicates the upper boundary of 90% confidence interval. Fisher’s exact test is applied to compare the samples above and within the confidence interval. **C** The predicted accuracy of random forest classifier based on leave-one-out validation for each batch (splitting training set: test set = 8:2, bootstrapping *n *= 20). **D** The top 15 most important OTUs identified by the random forest classifier. **E** The detected frequency of the top 15 most important OTUs in the four main batches, two-tailed Student’s *t*-test, FDR-corrected, *P < 0.05, **P < 0.01, ***P < 0.001. **F** The predicted probabilities of samples from batch S based on the optimized classifier established using the top 15 most important OTUs (top panel). Heatmap shows the relative abundance of these OTUs in batch S (bottom panel)

Although the gut microbiota of the wild and farmed croakers showed a high inter-batch variation, the overall divergence and non-stochastic distribution of most abundant OTUs suggest distinguishable and major deterministic microbial assembly patterns. We then tested the performance of the random forest classification under different ratios for the training and test sets. As shown in Fig. 5A, the average area-under-curve (AUC) value increases from 0.898 in the 5:5 sets to 0.943 in the 9:1 sets. From the 5:5 set to the 8:2 set, the accuracy of the farmed group is consistently lower than that of the wild group, which may be related to the higher inter-batch divergence and stronger stochastic assembly pattern in the farmed group.

To validate whether the stochastic assembly has a negative effect on the random forest classification, we calculated the average probability (> 50% for a correct assignment) of each sample via bootstrapping (*n* = 100, designating 5:5 of training: test for each batch). The accumulated relative abundance of the neutral OTUs and below-prediction OTUs were also determined for the farmed and wild samples, respectively (see Fig. 4C and D for the definition of the OTUs). As shown in Fig. 5B, the samples with high relative abundance of neutral OTUs and below-prediction OTUs (higher than the upper boundary of 90% confidence interval) are more likely to be poorly assigned (average probability < 50%) compared with the other samples (*P* < 0.05, Fisher’s exact test). Reasonably, the samples with a high abundance of below-prediction OTUs are likely dominated by few taxa, and processing a simple microbiota also may not sufficiently support the classification. The poor assignment of samples with a high abundance of stochastic OTUs supports the finding regarding the negative effect of stochastic microbial assembly on the random forest classification.

Then, we selected the 8:2 set in which all AUC values were higher than 0.9 (in ten replications). The results of the leave-one-out validation suggest that some batches (e.g., T and W) were likely assigned incorrectly (Fig. 5C), further indicating that good sample representability is a prerequisite for the good performance of the machine learning classifier. Moreover, as batch W was obtained from a remote geographical location with respect to most other batches, the low classification performance may be partially attributed to biogeography.

As suggested by the *k*-fold cross-validation (Fig. S10), 15 OTUs could generate the lowest error rate of prediction. Thus, the top 15 classifier OTUs that contributed to accurate classification were listed (Fig. 5D). A large proportion of the classifiers were core OTUs from the wild and farmed groups. *Psychrobacter* spp. were highly weighted in the algorithm, and its relative abundance was considered to be the strongest factor related positively to the predicted probabilities for the wild individuals. The optimized classifier was kept for downstream analysis. A wild batch S (*n* = 15), which was collected in the same bay as most of the farmed batches, was additionally tested. Notably, the frequency of the top 15 classifier OTUs was significantly lower in this batch than in the other wild batches (all *P* < 0.001, two-tailed Student’s *t*-test, Fig. 5E). Batch S was enriched with only two *Psychrobacter* OTUs, while most other OTUs were extremely low or missing (Fig. 5F). Interestingly, all of the predicting results were correct, although the probabilities were low (0.70 ± 0.09, Fig. 5F). To further validate the superiority of the machine learning algorithm, we clustered all samples based on Bray–Curtis distance for all, core, and 15 classifier OTUs (Fig. S11). Approximately 10% of the wild samples (23–24 in total, including 1–3 from batch S) were clustered with most farmed individuals (> 95%) for each of the samples regardless of the referring dataset. Surprisingly, the high proportion of these wild samples (> 90%) could be correctly assigned by the machine learning approach. These results indicate the robustness of the random forest classifier.

## Discussion

The machine learning classification of gut microbiota has been extensively used to predict host phenotypes in humans [67, 68]. A recent study reported the first attempt to apply the gut microbiome in fish host tracking in terms of habitat and taxonomy, and they found that habitat is a major factor for shaping the gut microbiome of various fish species [8]. However, the prediction accuracy of the machine learning algorithm was low for habitat at AUC < 0.8. For the first time, our study has validated the feasibility of utilizing gut microbiome for the robust host source tracking in fish. Meanwhile, the insufficient representativeness of training datasets leads to an overfitting and a failure of prediction of newly introduced samples [69]. In determining the divergence between wild and farmed fishes, most previous researchers collected a few or even single-batch samples, overlooking the potential high inter-batch divergence that has been observed by our study and potentially causing biases in profiling the taxonomic and functional features. In particular, our study found highly divergent core-taxa can for the samples from different batches and sources (Fig. 4A and B).

Obvious divergences between the gut microbiomes of the wild and farmed croakers were determined in terms of their alpha- and beta-diversity. An unexpected phenomenon is that the rectum bacterial diversity was lower for the wild individuals compared with the farmed samples (Fig. 2A). An apparent reason is the domination of the single genus *Psychrobacter* in wild individuals. Higher alpha diversity of gut microbiota triggered by simplified diets has been reported in fish [70–72]. For the beta-diversity, our results, which included those from NMDS and cluster analysis, did not support a clear cut between the gut microbiota of the wild and farmed fishes (Fig. 2A and C). It indicates that the common beta-diversity analysis cannot be directly applied to host source tracking. Also, determining the presence of certain marker taxa of microbiome (an approach adopted by a few previous studies in animal host tracking [73, 74]), would be insufficient because almost all major OTUs were shared by wild and farmed fishes, although they were divergently distributed (Fig. 2C). Therefore, the machine learning approach, which is essentially a supervised analysis to seek undefined and complex features related to a certain phenotype [75], was chosen for the objective of host source tracking.

Stochastic processes play a key role in shaping microbial assemblies in many environments [76, 77]. However, previous studies and our research indicate that deterministic processes usually play an important role in gut microbial assemblies in fishes, suggesting the presence of high niche selection stress on community structures [15, 17]. In the present study, despite the highly variable microbiota among the different batches, we found that almost all major or core OTUs deviated from the neutral model, indicating their underlying deterministic assembly pattern. A recent study revealed the lower contribution of neutral processes in the gut microbial assembly of wild Atlantic salmon compared with those in farmed individuals [10]. Our study also discovered a higher goodness-of-fit to the neutral model in farmed individuals than wild ones, suggesting a potential disconnection of host–microbe interaction in farming circumstances. As our results suggest, the functional capacity of the gut microbiome and isolates differ between the farmed and wild croakers. Thus, it is interesting to investigate whether the increase in goodness-of-fit to the neutral model for the farmed fishes is related to diet variation or other factors. More importantly, stochastic systems are intrinsically unfavorable for machine learning classification as they can generate classifiers established by false signals, e.g., *p*-hacking [64]. Despite the overall poor goodness-of-fit of the gut microbial assembly and major OTUs in both wild and farmed croakers, the results support the linking of a high proportion of stochastic OTUs to the errors in host source tracking (Fig. 5B). The successful host source tracking of the newly introduced batch S has verified the robustness of the classifier building based on the non-stochastic assembly pattern of the gut microbiota. Therefore, we recommend the evaluation and exclusion of the effect of stochastic events when applying machine learning to host source tracking based on microbiota.

Despite the good performance in discriminating between wild and farmed individuals, the microbiota-based classifier may have other untested problems in the practice of wild resource assessment, such as when discriminating the true wild fishes from those escaping from farming cages and artificial release of fry. The dynamics and profile of gut microbiota of the escaping fishes or the released fry have not been examined in the present study. The rapid shift (from days to a few months) of gut microbiota during domestication and diet change has been revealed in African cichlid, European seabass, grass carp, perch, etc. [78–81]. In carnivorous European seabass, mucosa-associated microbiota was found to be more stable than the corresponding digesta microbiota when shifting to plant-based diet [80]. Investigations pertaining to the effect of diet shift on the dynamics of gut microbiota in different intestinal locations may provide more basis for the practice of wild resource assessment of croaker.

Finally, although not the major aim of the present study, understanding the underlying mechanisms responsible for the divergence of gut microbiomes between the wild and farmed fishes can provide key information on improving aquaculture production [22]. Dysbiosis has been widely reported in aquaculture fishes fed with formulated feed [82]. Our study revealed possible dysbiosis in farmed croaker because potential pathogenic bacterial taxa, such as *Vibrio* spp., *Photobacterium* spp., etc., can be the core taxa, whereas they were less frequently and abundantly detected in the wild individuals. By contrast, the wild samples were dominated by *Psychrobacter* spp., which is widely detected in the gut of marine fish [5]. A few strains of this genus have been tested for their probiotic applications in fish diets [83, 84]. Whether the *Psychrobacter* strains from croakers can serve as probiotics for various applications, including improving stock enhancement (e.g., domestication by specialized diet before the release of fry) and diet-based gut microbiota regulation for farmed croakers, is worthy of further examination.

## Conclusions

The gut microbiome is not only closely related to the health and metabolism of hosts but also contains key information on the physiological and ecological circumstances of the hosts. Our study revealed the divergence of the gut microbiota and relevant functional profiles between wild and farmed croakers. With less biased datasets and non-stochastic patterns, we have verified for the first time that gut microbiota can be robustly applied to the tracking of host source even in carnivorous fish. A similar strategy can be applied to other fish species in need of discriminating source-unknown individuals.

## Supplementary Information

**Additional file 1: Figure S1.** Sampling sites and other information of the ten batches of croakers. **Figure S2.** Tissues of the gastrointestinal tract of the large yellow croaker and the section used in the gut microbiota analysis. **Figure S3.** Weighted UniFrac distance between or within batches. **Figure S4.** Beta-diversity analysis for the functional profile of gut microbiome in wild and farmed croakers. (A) Principal co-ordinates analysis of MetaCyc pathways based on Bray-Curtis distance, AMOVA test. (B) Positive correlation between pathway-level Bray-Curtis distance and OTU-level Bray-Curtis distance, 95% confidence interval. **Figure S5.** The relative abundance of top-level MetaCyc pathways in farmed and wild individuals. Wilcoxon test, * *P*<0.05, ** *P*<0.01, *** *P*<0.001. **Figure S6.** The major components of formulated feed used in rearing the farmed large yellow croakers. **Figure S7.** Maximum likelihood phylogenetic trees of representative isolates from Photobacterium (A) and Psycrobacter (B) based on 120 universal single-copy marker genes. Both trees are reconstructed under the JTT+G model. The 14 isolates are marked in bold, the brackets indicate the number of chitinase. The heatmaps show the average nucleotide identity (ANI) values among genome pairs. **Figure S8.** The predicted capability of carbohydrates utilization and organic acid production of *Photobacterium* (*n*=7) and *Psychrobacter* (*n *= 7) isolates. **Figure S9.** Photographs of biofilm formation assays of 14 isolates belonging to *Photobacterium* and *Psychrobacter*, bar=200 μm. **Figure S10.** Identification of the most important OTUs based on the 10-fold cross-validation. The lowest predicted error rate occurred at 15 OTUs and an optimized classifier was built using the top 15 most important OTUs according to the mean decrease accuracy. **Figure S11.** Clustering of all samples (*n *= 291) based on Euclidean distance with all (A), core (B), and 15 classifier OTUs (C). The Cluster I are dominant by farmed samples and the Cluster II are dominant by wild samples. The number present in the brackets indicate number of wild samples and farmed samples in each cluster. The pie charts show the proportion of correctly predicted wild samples which were clustered in cluster I using optimized random forest classifier.

## Data Availability

The sequencing datasets generated during the current study are available in the NCBI database. The 16S rRNA gene datasets were deposited in the Sequence Read Archive under accession number PRJNA679381. The genome sequences of *Photobacterium* and *Psychrobacter* strains in this study have been deposited in the NCBI database under the accession number PRJNA678775.

## Abbreviations

AMOVA: Analysis of molecular variance
AUC: Area-under-curve
βNTI: Beta nearest-taxon index
CAZyme: Carbohydrate-active enzyme
GHs: Glycoside hydrolases
GTs: Glycosyltransferases
NTI: Net relatedness index
NMDS: Nonmetric multidimensional scaling
OTU: Operational taxonomic unit
qPCR: Quantitative PCR

## References

[1] Clements KD, Angert ER, Montgomery WL, Choat JH (2014). Intestinal microbiota in fishes: what's known and what's not. Mol Ecol.

[2] Lee WJ, Hase K (2014). Gut microbiota-generated metabolites in animal health and disease. Nat Chem Biol.

[3] Ley RE, Hamady M, Lozupone C, Turnbaugh PJ, Ramey RR, Bircher JS (2008). Evolution of mammals and their gut microbes. Science..

[4] McKenney EA, O’Connell TM, Rodrigo A, Yoder AD (2018). Feeding strategy shapes gut metagenomic enrichment and functional specialization in captive lemurs. Gut Microbes.

[5] Egerton S, Culloty S, Whooley J, Stanton C, Ross RP (2018). The gut microbiota of marine fish. Front Microbiol.

[6] Ghanbari M, Kneifel W, Domig KJ (2015). A new view of the fish gut microbiome: advances from next-generation sequencing. Aquaculture..

[7] Wang AR, Ran C, Ringo E, Zhou ZG (2018). Progress in fish gastrointestinal microbiota research. Rev Aquac.

[8] Kim PS, Shin NR, Lee JB, Kim MS, Whon TW, Hyun DW (2021). Host habitat is the major determinant of the gut microbiome of fish. Microbiome..

[9] Kormas KA, Meziti A, Mente E, Frentzos A (2014). Dietary differences are reflected on the gut prokaryotic community structure of wild and commercially reared sea bream (*Sparus aurata*). Microbiologyopen..

[10] Llewellyn MS, McGinnity P, Dionne M, Letourneau J, Thonier F, Carvalho GR (2016). The biogeography of the Atlantic salmon (*Salmo salar*) gut microbiome. ISME J.

[11] Baldo L, Pretus JL, Riera JL, Musilova Z, Nyom ARB, Salzburger W (2017). Convergence of gut microbiotas in the adaptive radiations of African cichlid fishes. ISME J.

[12] Miyake S, Ngugi DK, Stingl U (2015). Diet strongly influences the gut microbiota of surgeonfishes. Mol Ecol.

[13] Heys C, Cheaib B, Busetti A, Kazlauskaite R, Maier L, Sloan WT (2020). Neutral processes dominate microbial community assembly in Atlantic salmon, *Salmo salar*. Appl Environ Microbiol.

[14] Roeselers G, Mittge EK, Stephens WZ, Parichy DM, Cavanaugh CM, Guillemin K (2011). Evidence for a core gut microbiota in the zebrafish. ISME J.

[15] Yan Q, Li J, Yu Y, Wang J, He Z, Van Nostrand JD (2016). Environmental filtering decreases with fish development for the assembly of gut microbiota. Environ Microbiol.

[16] Burns AR, Stephens WZ, Stagaman K, Wong S, Rawls JF, Guillemin K (2016). Contribution of neutral processes to the assembly of gut microbial communities in the zebrafish over host development. ISME J.

[17] Razak SA, Scribner KT (2020). Ecological and ontogenetic components of larval lake sturgeon gut microbiota assembly, successional dynamics, and ecological evaluation of neutral community processes. Appl Environ Microbiol.

[18] Stagaman K, Burns AR, Guillemin K, Bohannan BJ (2017). The role of adaptive immunity as an ecological filter on the gut microbiota in zebrafish. ISME J.

[19] Rawls JF, Mahowald MA, Ley RE, Gordon JI (2006). Reciprocal gut microbiota transplants from zebrafish and mice to germ-free recipients reveal host habitat selection. Cell..

[20] Kim DH, Kim DY (2013). Microbial diversity in the intestine of olive flounder (*Paralichthys olivaceus*). Aquaculture..

[21] Holben WE, Williams P, Saarinen M, Särkilahti L, Apajalahti JH (2002). Phylogenetic analysis of intestinal microflora indicates a novel *mycoplasma* phylotype in farmed and wild salmon. Microb Ecol.

[22] Limborg MT, Alberdi A, Kodama M, Roggenbuck M, Kristiansen K, Gilbert MTP (2018). Applied hologenomics: feasibility and potential in aquaculture. Trends Biotechnol.

[23] Ramírez C, Romero J (2017). The microbiome of *Seriola lalandi* of wild and aquaculture origin reveals differences in composition and potential function. Front Microbiol.

[24] Ramírez C, Coronado J, Silva A, Romero J (2018). *Cetobacterium* is a major component of the microbiome of giant Amazonian fish (*Arapaima gigas*) in Ecuador. Animals..

[25] Johansen LH, Jensen I, Mikkelsen H, Bjørn PA, Jansen P, Bergh Ø (2011). Disease interaction and pathogens exchange between wild and farmed fish populations with special reference to Norway. Aquaculture..

[26] Einum S, Fleming I (1997). Genetic divergence and interactions in the wild among native, farmed and hybrid Atlantic salmon. J Fish Biol.

[27] Brigante R, Lem A (2001). Price interaction between aquaculture and fishery.

[28] Bjørndal T, Guillen J (2017). Market integration between wild and farmed seabream and seabass in Spain. Appl Econ.

[29] Jardine LB, Burt MDB, Arp PA, Diamond AW (2009). Mercury comparisons between farmed and wild Atlantic salmon (*Salmo salar* L.) and Atlantic cod (*Gadus morhua* L.). Aquac Res.

[30] Karlsson S, Diserud OH, Fiske P, Hindar K. Widespread genetic introgression of escaped farmed Atlantic salmon in wild salmon populations. ICES J Mar Sci. 2016;73:2488–98.

[31] Chen S, Su Y, Hong W (2018). Aquaculture of the large yellow croaker. Aquaculture in China: success stories and modern trends.

[32] BOF, NFTEC, CSF. China fishery statistical yearbook. China: Fisheries Agency of China Agriculture Ministry, China, 2020.

[33] Liu M, De Mitcheson YS (2008). Profile of a fishery collapse: why mariculture failed to save the large yellow croaker. Fish Fish.

[34] Wang L, Shi X, Su Y, Meng Z, Lin H (2012). Loss of genetic diversity in the cultured stocks of the large yellow croaker, *Larimichthys crocea*, revealed by microsatellites. Int J Mol Sci.

[35] Zhang QY, Hong WS, Yang SY, Liu M (2011). Discussion on the division of geographic populations for the large yellow croaker (*Larimichthys crocea*). Modern Fish Inform.

[36] Eraslan G, Avsec Ž, Gagneur J, Theis FJ (2019). Deep learning: new computational modelling techniques for genomics. Nat Rev Genet.

[37] Roguet A, Eren AM, Newton RJ, McLellan SL (2018). Fecal source identification using random forest. Microbiome..

[38] Kozich JJ, Westcott SL, Baxter NT, Highlander SK, Schloss PD (2013). Development of a dual-index sequencing strategy and curation pipeline for analyzing amplicon sequence data on the MiSeq Illumina sequencing platform. Appl Environ Microbiol.

[39] MacConaill LE, Burns RT, Nag A, Coleman HA, Slevin MK, Giorda K (2018). Unique, dual-indexed sequencing adapters with UMIs effectively eliminate index cross-talk and significantly improve sensitivity of massively parallel sequencing. BMC Genomics.

[40] Muyzer G, Hottentrager S, Teske A, Wawer C (1996). Denaturing gradient gel electrophoresis of PCR-amplified 16S rDNA. A new molecular approach to analyze the genetic diversity of mixed microbial communities. Microbiol Ecol Mange.

[41] Bolger A, Lohse M, Usadel B (2014). Trimmomatic: a flexible trimmer for Illumina sequence data. Bioinformatics..

[42] Edgar RC. UNOISE2: improved error-correction for Illumina 16S and ITS amplicon sequencing. BioRxiv. 2016;081257.

[43] Thompson LR, Sanders JG, McDonald D, Amir A, Ladau J, Locey KJ (2017). A communal catalogue reveals Earth’s multiscale microbial diversity. Nature..

[44] Lozupone C, Lladser ME, Knights D, Stombaugh J, Knight R (2011). UniFrac: an effective distance metric for microbial community comparison. ISME J.

[45] Yoon SH, Ha SM, Kwon S, Lim J, Kim Y, Seo H (2017). Introducing EzBioCloud: a taxonomically united database of 16S rRNA gene sequences and whole-genome assemblies. Int J Syst Evol Microbiol.

[46] Mukuta Y, Harada T. Probabilistic partial canonical correlation analysis. In ICML. 2014;32:1449–57.

[47] Kolde R. pheatmap: pretty heatmaps. R package version 1.0.10. 2018.

[48] Oksanen J, Blanchet F, Friendly M, Kindt R, Legendre P, McGlinn D, et al. Vegan: community ecology package, R package version 2.5–3. 2018.

[49] Sloan WT, Lunn M, Woodcock S, Head IM, Nee S, Curtis TP (2006). Quantifying the roles of immigration and chance in shaping prokaryote community structure. Environ Microbiol.

[50] Kembel SW, Cowan PD, Helmus MR, Cornwell WK, Morlon H, Ackerly DD (2010). Picante: R tools for integrating phylogenies and ecology. Bioinformatics.

[51] Russel J (2021). MicEco: various functions for microbial community data. R package version 0.9.15.

[52] Douglas GM, Maffei VJ, Zaneveld JR, Yurgel SN, Brown JR, Taylor CM (2020). PICRUSt2 for prediction of metagenome functions. Nat Biotechnol.

[53] Fernandes AD, Reid JN, Macklaim JM, McMurrough TA, Edgell DR, Gloor GB (2014). Unifying the analysis of high-throughput sequencing datasets: characterizing RNA-seq, 16S rRNA gene sequencing and selective growth experiments by compositional data analysis. Microbiome..

[54] Bankevich A, Nurk S, Antipov D, Gurevich AA, Dvorkin M, Kulikov AS (2012). SPAdes: a new genome assembly algorithm and its applications to single-cell sequencing. J Comput Biol.

[55] Hyatt D, Chen G-L, LoCascio PF, Land ML, Larimer FW, Hauser LJ (2010). Prodigal: prokaryotic gene recognition and translation initiation site identification. BMC Bioinformatics.

[56] Zhang H, Yohe T, Huang L, Entwistle S, Wu P, Yang Z (2018). dbCAN2: a meta server for automated carbohydrate-active enzyme annotation. Nucleic Acids Res.

[57] Petersen TN, Brunak S, Von Heijne G, Nielsen H (2011). SignalP 4.0: discriminating signal peptides from transmembrane regions. Nat Methods.

[58] Lin H, Chen W, Ding H (2013). AcalPred: a sequence-based tool for discriminating between acidic and alkaline enzymes. PLoS One.

[59] Shaffer M, Borton MA, McGivern BB, Zayed AA, La Rosa SL, Solden LM (2020). DRAM for distilling microbial metabolism to automate the curation of microbiome function. Nucleic Acids Res.

[60] O'Toole GA (2001). Microtiter dish biofilm formation assay. J Vis Exp.

[61] Breiman L (2001). Random forests. Mach Learn.

[62] Robin X, Turck N, Hainard A, Tiberti N, Lisacek F, Sanchez J-C (2011). pROC: an open-source package for R and S+ to analyze and compare ROC curves. BMC Bioinformatics.

[63] Kuhn M. Building predictive models in R using the caret package. J Stat Softw. 2008;28:1–26.

[64] Xu J, Chen J, Tian F, Xu Z. Summer diet composition and feeding ecology of large yellow croaker (*Larimichthys crocea*) in Guanjing Yang. J Fish Sci China. 2012;19:94–104 (in Chinese).

[65] Wu C, Zhang D, Kan M, Lv Z, Zhu A, Su Y (2014). The draft genome of the large yellow croaker reveals well-developed innate immunity. Nat Commun.

[66] Stegen JC, Lin X, Fredrickson JK, Chen X, Kennedy DW, Murray CJ (2013). Quantifying community assembly processes and identifying features that impose them. ISME J.

[67] Subramanian S, Huq S, Yatsunenko T, Haque R, Mahfuz M, Alam MA (2014). Persistent gut microbiota immaturity in malnourished Bangladeshi children. Nature..

[68] Kong F, Hua Y, Zeng B, Ning R, Li Y, Zhao J (2016). Gut microbiota signatures of longevity. Curr Biol.

[69] Schat E, van de Schoot R, Kouw WM, Veen D, Mendrik AM (2020). The data representativeness criterion: predicting the performance of supervised classification based on data set similarity. PLoS One.

[70] Li H, Qu J, Li T, Wirth S, Zhang Y, Zhao X (2018). Diet simplification selects for high gut microbial diversity and strong fermenting ability in high-altitude pikas. Appl Microbiol Biotechnol.

[71] Bolnick DI, Snowberg LK, Hirsch PE, Lauber CL, Org E, Parks B (2014). Individual diet has sex-dependent effects on vertebrate gut microbiota. Nat Commun.

[72] Li P, Wu G (2018). Roles of dietary glycine, proline, and hydroxyproline in collagen synthesis and animal growth. Amino Acids.

[73] Lee JE, Lee S, Sung J, Ko G (2011). Analysis of human and animal fecal microbiota for microbial source tracking. ISME J.

[74] Zhang Y, Wu R, Lin K, Wang Y, Lu J (2020). Performance of host-associated genetic markers for microbial source tracking in China. Water Res.

[75] Namkung J (2020). Machine learning methods for microbiome studies. J Microbiol.

[76] Roguet A, Laigle GS, Therial C, Bressy A, Soulignac F, Catherine A (2015). Neutral community model explains the bacterial community assembly in freshwater lakes. FEMS Microbiol Ecol.

[77] Chen W, Ren K, Isabwe A, Chen H, Liu M, Yang J (2019). Stochastic processes shape microeukaryotic community assembly in a subtropical river across wet and dry seasons. Microbiome..

[78] Baldo L, Riera JL, Tooming-Klunderud A, Albà MM, Salzburger W (2015). Gut microbiota dynamics during dietary shift in eastern African cichlid fishes. PLoS One.

[79] Serra CR, Oliva-Teles A, Enes P, Tavares F (2021). Gut microbiota dynamics in carnivorous European seabass (*Dicentrarchus labrax*) fed plant-based diets. Sci Rep.

[80] Hao YT, Wu SG, Xiong F, Tran NT, Jakovlić I, Zou H (2017). Succession and fermentation products of grass carp (*Ctenopharyngodon idellus*) hindgut microbiota in response to an extreme dietary shift. Front Microbiol.

[81] Zha Y, Eiler A, Johansson F, Svanbäck R (2018). Effects of predation stress and food ration on perch gut microbiota. Microbiome..

[82] Infante-Villamil S, Huerlimann R, Jerry DR. Microbiome diversity and dysbiosis in aquaculture. Rev Aquac. 2020;13:1077–96.

[83] Sun YZ, Yang HL, Ma RL, Zhang CX, Lin WY (2011). Effect of dietary administration of *Psychrobacter* sp. on the growth, feed utilization, digestive enzymes and immune responses of grouper *Epinephelus coioides*. Aquac Nutr.

[84] Makled SO, Hamdan AM, El-Sayed A-FM, Hafez EE (2017). Evaluation of marine psychrophile, *Psychrobacter namhaensis* SO89, as a probiotic in Nile tilapia (*Oreochromis niloticus*) diets. Fish Shellfish Immun.

