# Supplementary material for: Robust host source tracking building on the divergent and non-stochastic assembly of gut microbiomes in wild and farmed large yellow croaker
Source: Microbiome. 2022 Jan 26;10:18. doi: 10.1186/s40168-021-01214-7 (PMC8790850; doi:10.1186/s40168-021-01214-7)
Supplement: Supplementary file 2 — Additional file 1: Figure S1. Sampling sites and other information of the ten batches of croakers. Figure S2. Tissues of the gastrointestinal tract of the large yellow croaker and the section used in the gut microbiota analysis. Figure S3. Weighted UniFrac distance between or within batches. Figure S4. Beta-diversity analysis for the functional profile of gut microbiome in wild and farmed croakers. (A) Principal co-ordinates analysis of MetaCyc pathways based on Bray-Curtis distance, AMOVA test. (B) Positive correlation between pathway-level Bray-Curtis distance and OTU-level Bray-Curtis distance, 95% confidence interval. Figure S5. The relative abundance of top-level MetaCyc pathways in farmed and wild individuals. Wilcoxon test, * P<0.05, ** P<0.01, *** P<0.001. Figure S6. The major components of formulated feed used in rearing the farmed large yellow croakers. Figure S7. Maximum likelihood phylogenetic trees of representative isolates from Photobacterium (A) and Psycrobacter (B) based on 120 universal single-copy marker genes. Both trees are reconstructed under the JTT+G model. The 14 isolates are marked in bold, the brackets indicate the number of chitinase. The heatmaps show the average nucleotide identity (ANI) values among genome pairs. Figure S8. The predicted capability of carbohydrates utilization and organic acid production of Photobacterium (n=7) and Psychrobacter (n = 7) isolates. Figure S9. Photographs of biofilm formation assays of 14 isolates belonging to Photobacterium and Psychrobacter, bar=200 μm. Figure S10. Identification of the most important OTUs based on the 10-fold cross-validation. The lowest predicted error rate occurred at 15 OTUs and an optimized classifier was built using the top 15 most important OTUs according to the mean decrease accuracy. Figure S11. Clustering of all samples (n = 291) based on Euclidean distance with all (A), core (B), and 15 classifier OTUs (C). The Cluster I are dominant by farmed samples and the Cluster II are d [file 40168_2021_1214_MOESM2_ESM.docx]

*Supplementary materials for*

**Robust host source tracking building on the divergent and non-stochastic assembly of gut microbiomes in wild and farmed large yellow croaker**

**Jun Zhu^1, #^, Hao Li^1, #^, Ze-Zhou Jing^2, #^, Wei Zheng^1^, Yuan-Rong Luo^3^, Shi-Xi Chen^2, *^, Feng Guo^1, 4, *^**

1. School of Life Sciences, Southern Marine Science and Engineering Guangdong Laboratory (Zhuhai), Xiamen University, Xiamen, China
2. State-Province Joint Engineering Laboratory of Marine Bioproducts and Technology, College of Ocean and Earth Sciences, Xiamen University, Xiamen, China
3. Key Laboratory of the Ministry of Education for Coastal and Wetland Ecosystems, Xiamen University, Xiamen, China
4. Key Laboratory of Fujian Provincial University for Microorganism Resource, Xiamen, China

^#^These authors contribute equally

^*^Corresponding author.

Email: fguobio@xmu.edu.cn; chenshixi@xmu.edu.cn


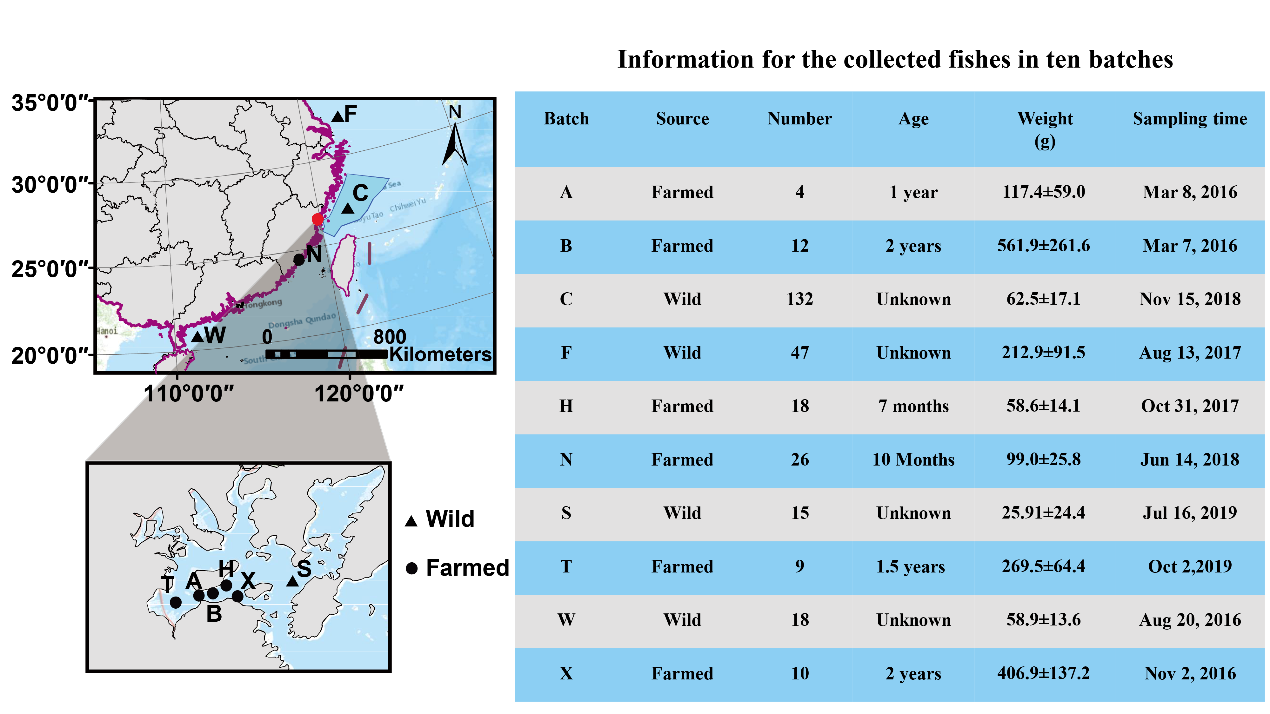
Figure S1 Sampling sites and other information of the ten batches of croakers.

**
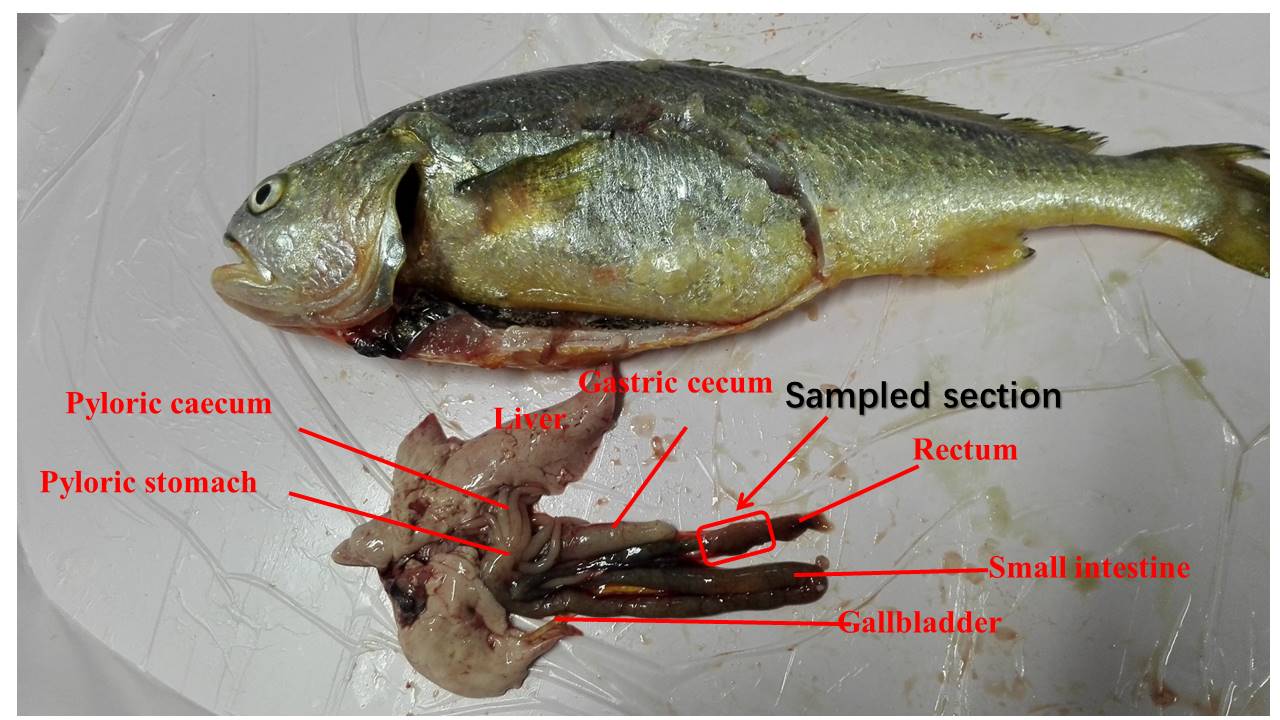
**

Figure S2 Tissues of the gastrointestinal tract of the large yellow croaker and the section used in the gut microbiota analysis.


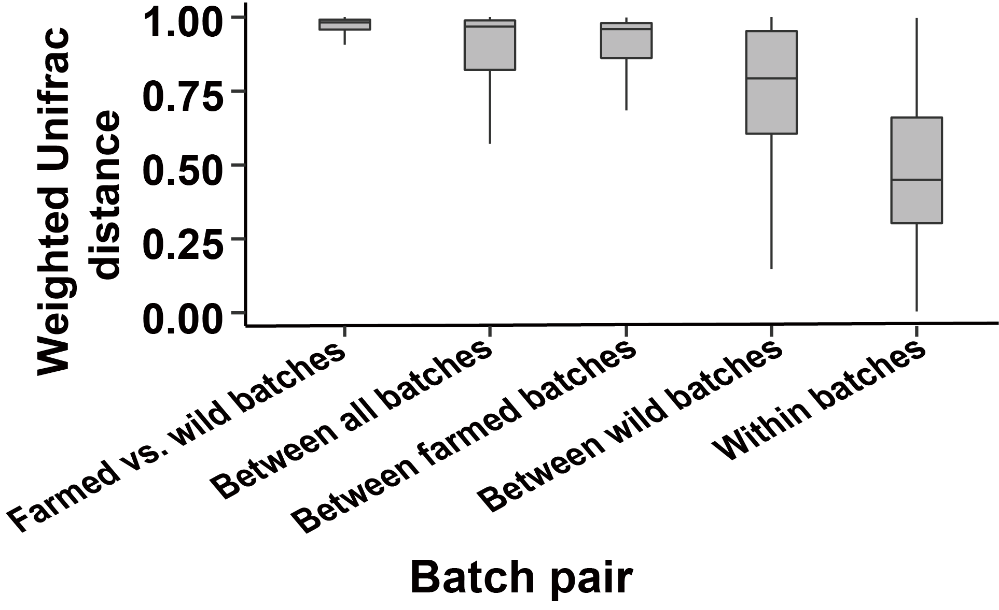
 Figure S3 Weighted UniFrac distance between or within batches.


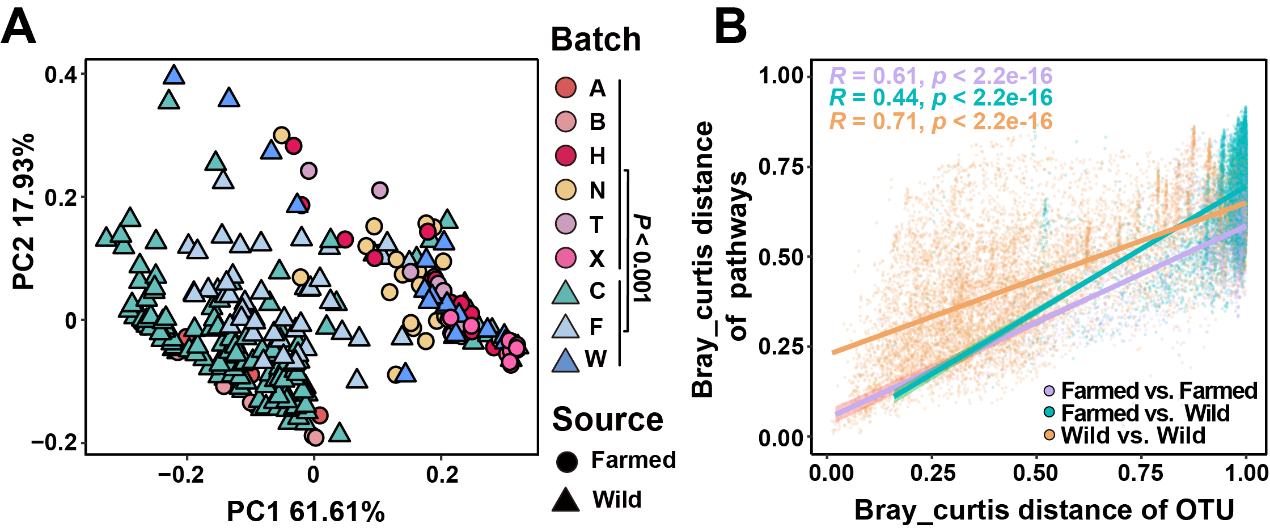
Figure S4 Beta-diversity analysis for the functional profile of gut microbiome in wild and farmed croakers. (A) Principal co-ordinates analysis of MetaCyc pathways based on Bray-Curtis distance, AMOVA test. (B) Positive correlation between pathway-level Bray-Curtis distance and OTU-level Bray-Curtis distance, 95% confidence interval.


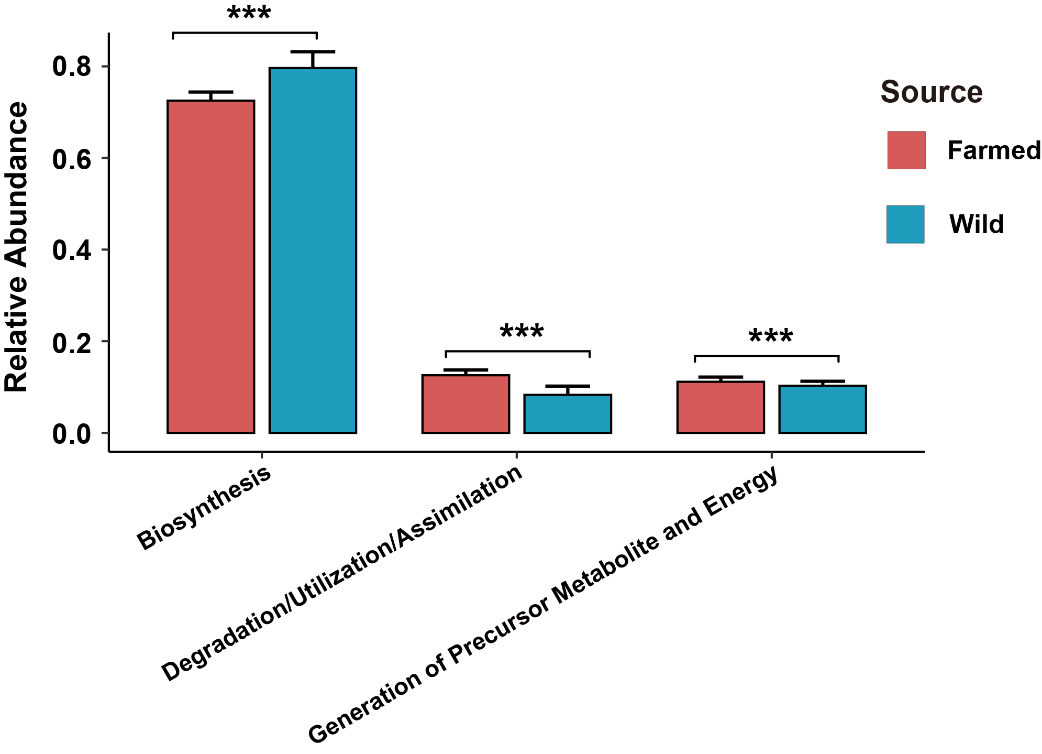


Figure S5 The relative abundance of top-level MetaCyc pathways in farmed and wild individuals. Wilcoxon test, ^*^ *P*<0.05, ^**^ *P*<0.01, ^***^ *P*<0.001.


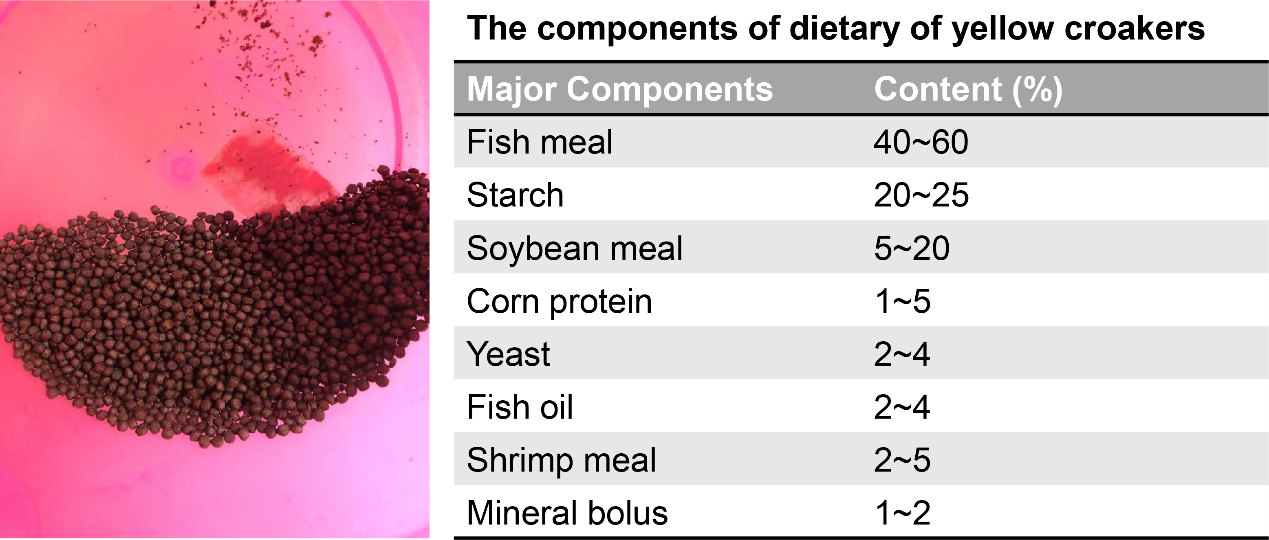


Figure S6 The major components of formulated feed used in rearing the farmed large yellow croakers.


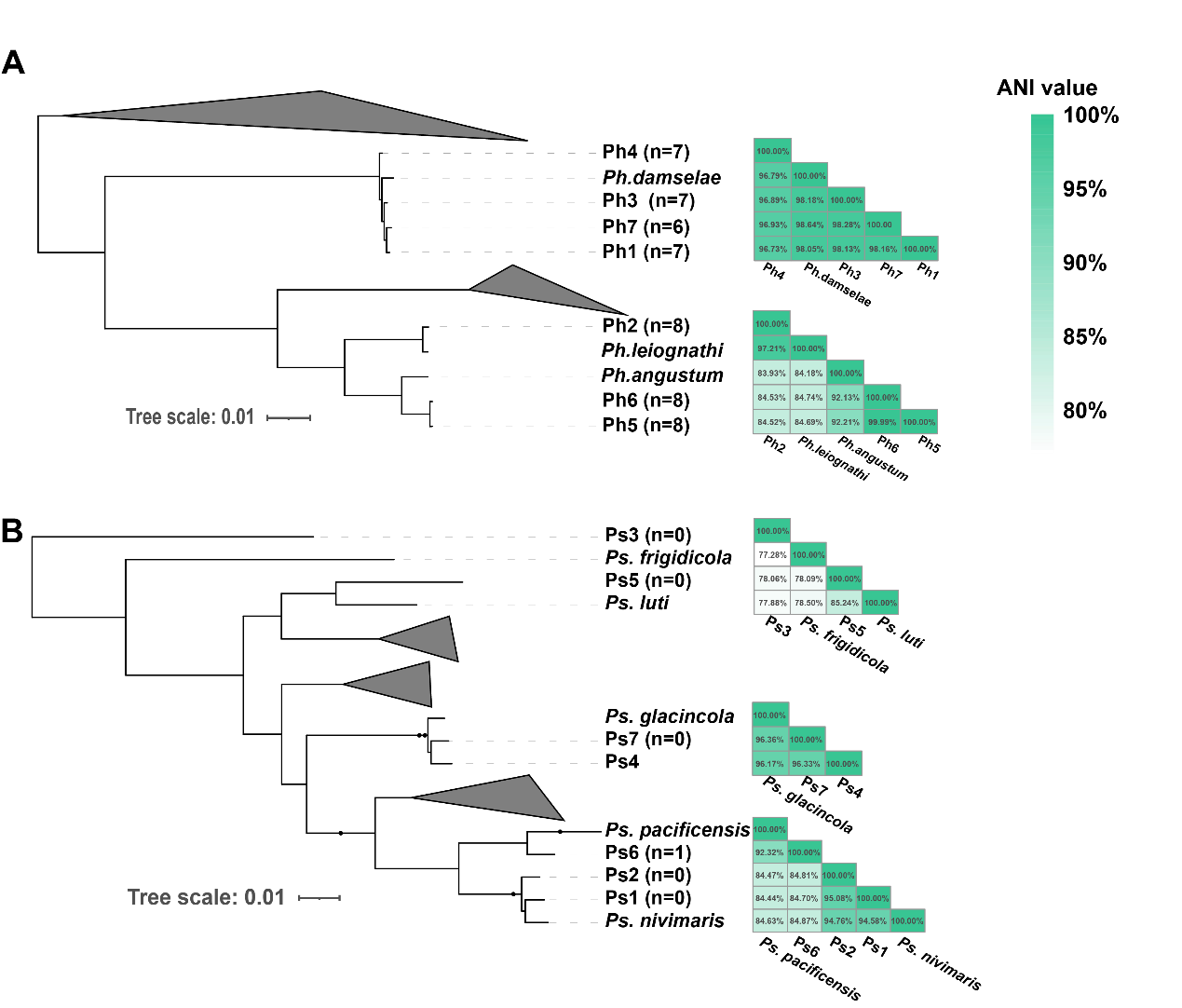


Figure S7 Maximum likelihood phylogenetic trees of representative isolates from *Photobacterium* (A) and *Psycrobacter* (B) based on 120 universal single-copy marker genes. Both trees are reconstructed under the JTT+G model. The 14 isolates are marked in bold, the brackets indicate the number of chitinase. The heatmaps show the average nucleotide identity (ANI) values among genome pairs.


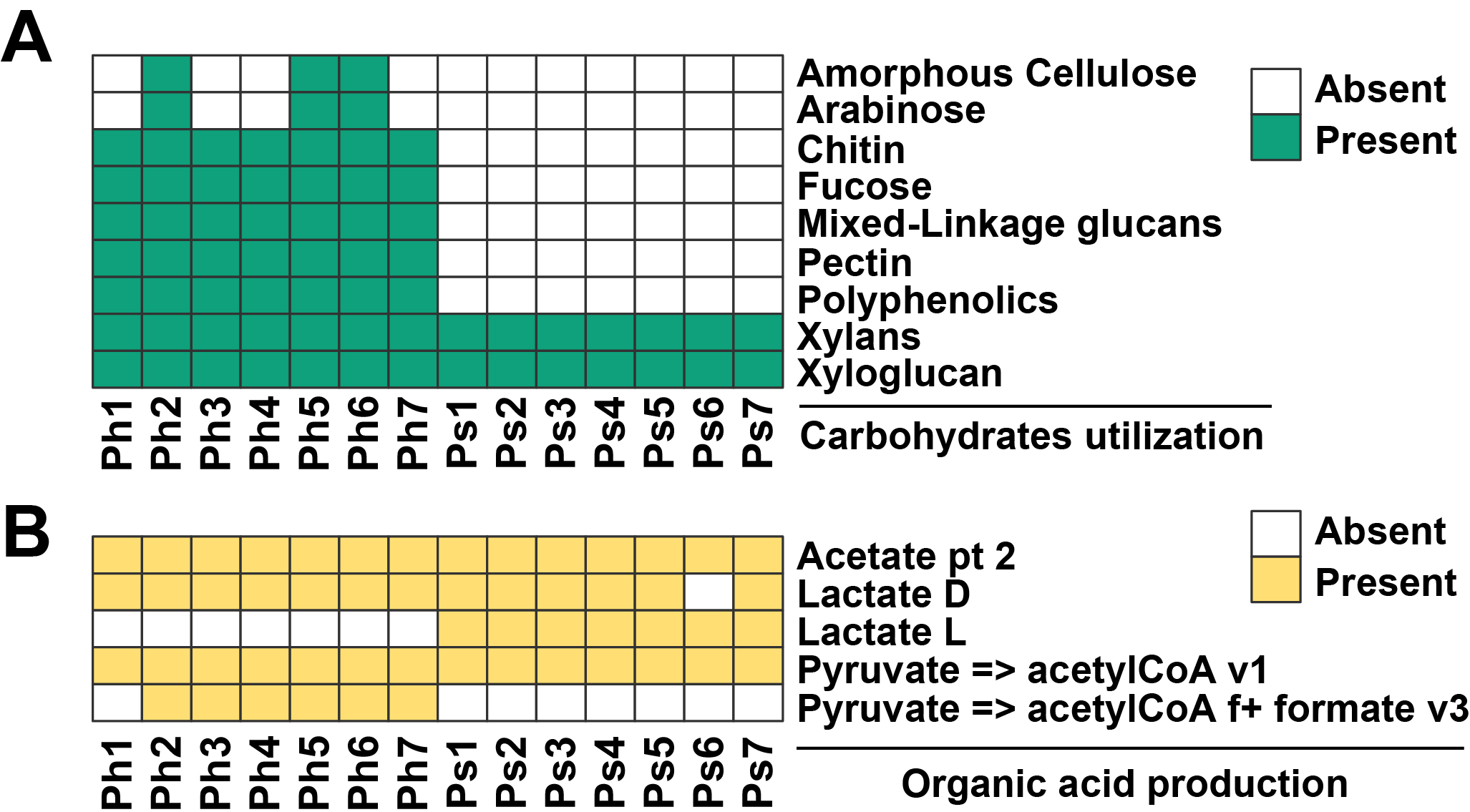


Figure S8 The predicted capability of carbohydrates utilization and organic acid production of *Photobacterium* (n=7) and *Psychrobacter* (n=7) isolates.


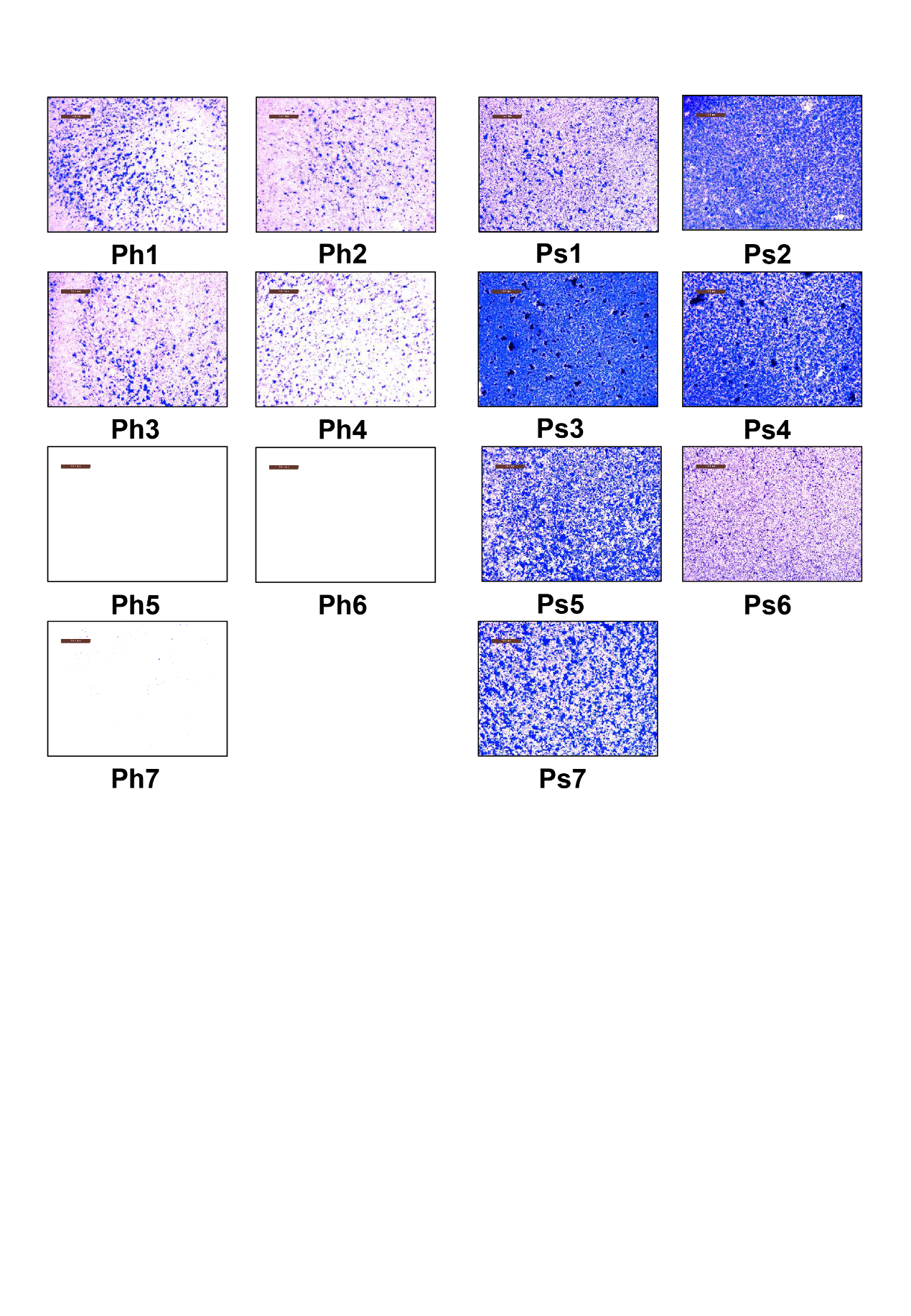


Figure S9 Photographs of biofilm formation assays of 14 isolates belonging to *Photobacterium* and *Psychrobacter*, bar=200 μm.


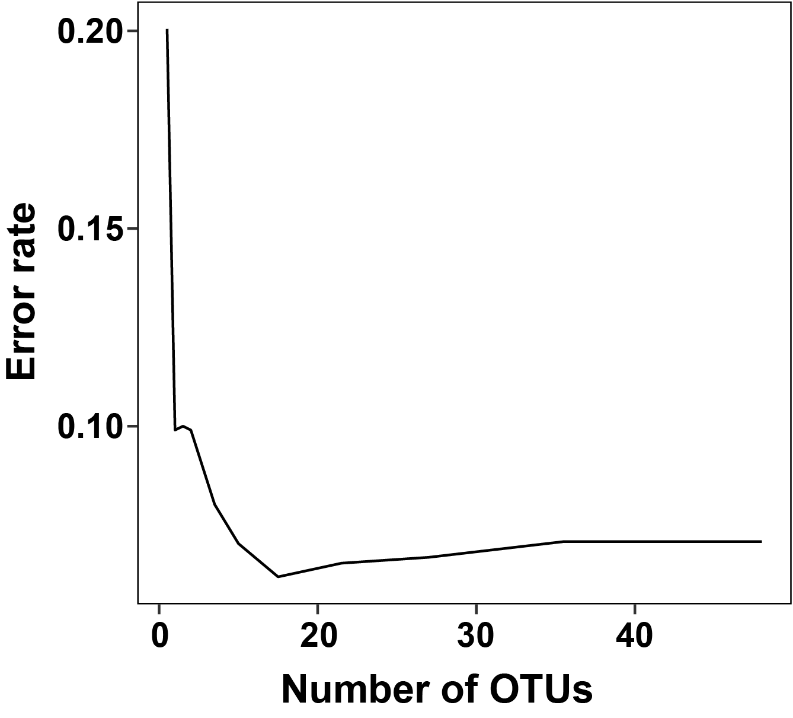


Figure S10 Identification of the most important OTUs based on the 10-fold cross-validation. The lowest predicted error rate occurred at 15 OTUs and an optimized classifier was built using the top 15 most important OTUs according to the mean decrease accuracy.


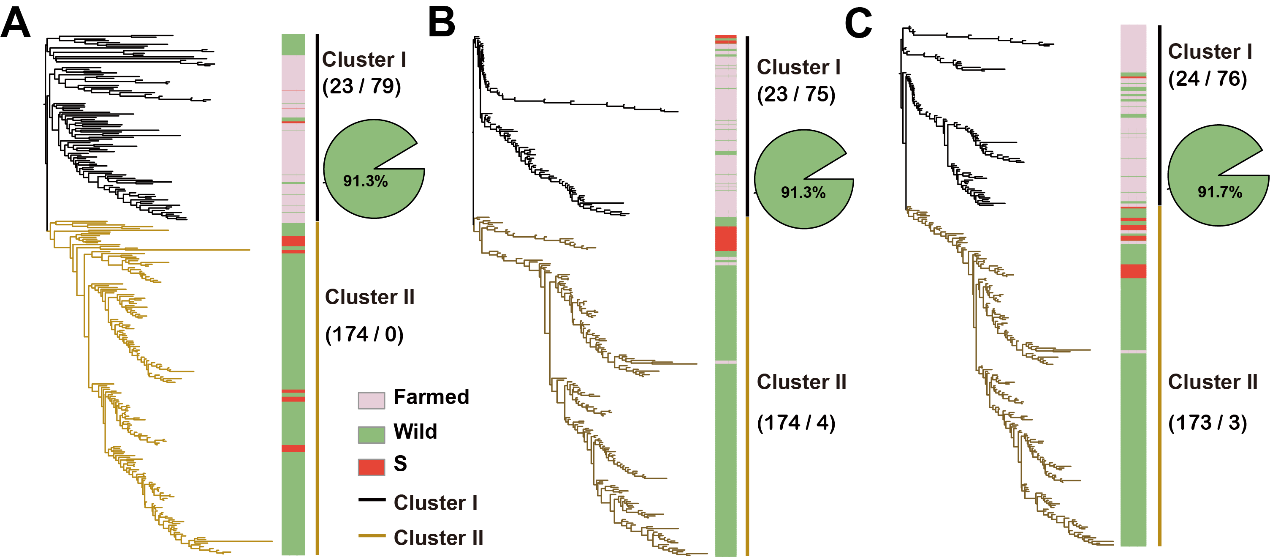


Figure S11 Clustering of all samples (*n*=291) based on Euclidean distance with all (A), core (B), and 15 classifier OTUs (C). The Cluster I are dominant by farmed samples and the Cluster II are dominant by wild samples. The number present in the brackets indicate number of wild samples and farmed samples in each cluster. The pie charts show the proportion of correctly predicted wild samples which were clustered in cluster I using optimized random forest classifier.
